# Commentary: SGLT2is vs. GLP1RAs reduce cardiovascular and all-cause mortality

**DOI:** 10.3389/fcvm.2022.987025

**Published:** 2022-08-04

**Authors:** Lixin Du, Jiao Qin, Dengchuan Wang, Yunhui Zhao, Ning Xu, Chaowen Wu

**Affiliations:** ^1^Department of Medical Imaging, Shenzhen Longhua District Central Hospital, Shenzhen, China; ^2^Office of Medical Ethics, Shenzhen Longhua District Central Hospital, Shenzhen, China; ^3^Department of Endocrinology, Shenzhen Longhua District Central Hospital, Shenzhen, China

**Keywords:** cardiovascular, renal, death, type 2 diabetes, SGLT 2 inhibitors, GLP-1RAs

## Introduction

By implementing a meta-analysis (1) based on nine large cohort studies (2–10) directly comparing cardiovascular and renal endpoints between sodium-glucose cotransporter 2 inhibitors (SGLT2is) and glucagon-like peptide 1 receptor agonists (GLP1RAs) in patients with type 2 diabetes (T2D), Qiu et al. (1) identified an interesting and clinically relevant finding that compared with GLP1RAs, SGLT2is were associated with significantly decreased risks of cardiovascular death (CVDH) [hazard ratio (HR) 0.82, 95% confidence interval (CI) 0.68–0.99] and all-cause mortality (ACM) (HR 0.92, 95% CI 0.85–0.99). Nowadays, nine new relevant cohort studies (11–19) are available and yield some inconsistent findings. For example, the studies by Fu et al. (11) and Ueda et al. (12) show similar risk of CVDH between SGLT2is and GLP1RAs, while the studies by Alkabbani et al. (13) and Tang et al. (14) show similar risk of ACM. Moreover, in Qiu's et al. (1) meta-analysis only a few of studies were incorporated when some endpoints were assessed, such as composite kidney outcome (CKO). This also suggests the necessity of performing an updated meta-analysis in which more studies could be included in order to provide greater statistical power. Hence, we carried out an updated meta-analysis and aimed at confirming and updating the findings of Qiu's et al. (1) meta-analysis.

## Methods

We conducted this updated meta-analysis in accordance with the statement of Preferred Reporting Items for Systematic Reviews and Meta-Analyses (PRISMA) (20).

The studies eligible for inclusion were cohort studies directly comparing SGLT2is and GLP1RAs in cardio-renal endpoints in T2D patients. Seven endpoints of interest are the same as those in Qius et al. (1) meta-analysis: stroke, hospitalization for heart failure (HHF), CKO, major adverse cardiovascular events (MACE), myocardial infarction (MI), CVDH, and ACM. CKO was defined as a composite of eGFR 50% reduction or lower than 60 ml/min/1.73 m^2^, micro- or macro-albuminuria, renal failure, dialysis, renal transplantation, or renal death; and MACE was defined as a composite of CVDH, MI, or stroke. We searched PubMed, Embase, and Web of science from inception date to May 2022. The whole search expression is (using PubMed as an example): [“Diabetes Mellitus, Type 2” (Mesh) OR “diabetes” (all fields)] AND [Sodium-Glucose Transporter 2 Inhibitors (MH) OR “Sodium glucose transporter 2 inhibitor^*^” (TIAB) OR “Sodium glucose cotransporter 2 inhibitor^*^” (TIAB) OR “Sodium glucose co-transporter 2 inhibitor^*^” (TIAB) OR SGLT^*^(TIAB) OR Gliflozin^*^(tiab) OR “Empagliflozin” (tiab) OR “Dapagliflozin” (tiab) OR “Canagliflozin” (tiab) OR “ertugliflozin” (tiab) OR “sotagliflozin” (tiab)] AND [“glucagon-like peptide 1 receptor agonist^*^” (TIAB) OR “GLP1^*^” (TIAB) OR lixisenatide (TIAB) OR liraglutide (TIAB) OR semaglutide (TIAB) OR exenatide (TIAB) OR albiglutide (TIAB) OR dulaglutide (TIAB) OR Efpeglenatide (TIAB)] AND [“cardiovascular” (tiab) OR “cardiac” (tiab) OR “heart failure” (tiab) OR “myocardial infarction” (TIAB) OR stroke (tiab) OR “MACE” (tiab) OR “Kidney” (tiab) OR “renal” (tiab) OR “CKD” (tiab) OR “ESRD” (tiab) OR “ESKD” (tiab) OR “cardiorenal” (tiab) OR “death” (tiab) OR “mortality” (tiab)] AND [“cohort study” (tiab) OR “observational study” (tiab) OR “real world” (tiab) OR “real-world” (tiab)].

The outcome data were extracted from included studies independently by two authors, and any discrepancies between them were solved by discussing with a third author. Just like Qiu et al. (1), we performed random-effects meta-analyses based on HRs and 95% CIs to derive conservative estimates for the relative effectiveness of SGLT2is and GLP1RAs. *P* <0.05 was considered as statistical significance. All statistical analyses were done using Stata/MP (Version 16.0).

## Findings and implications

**Figure 1:**
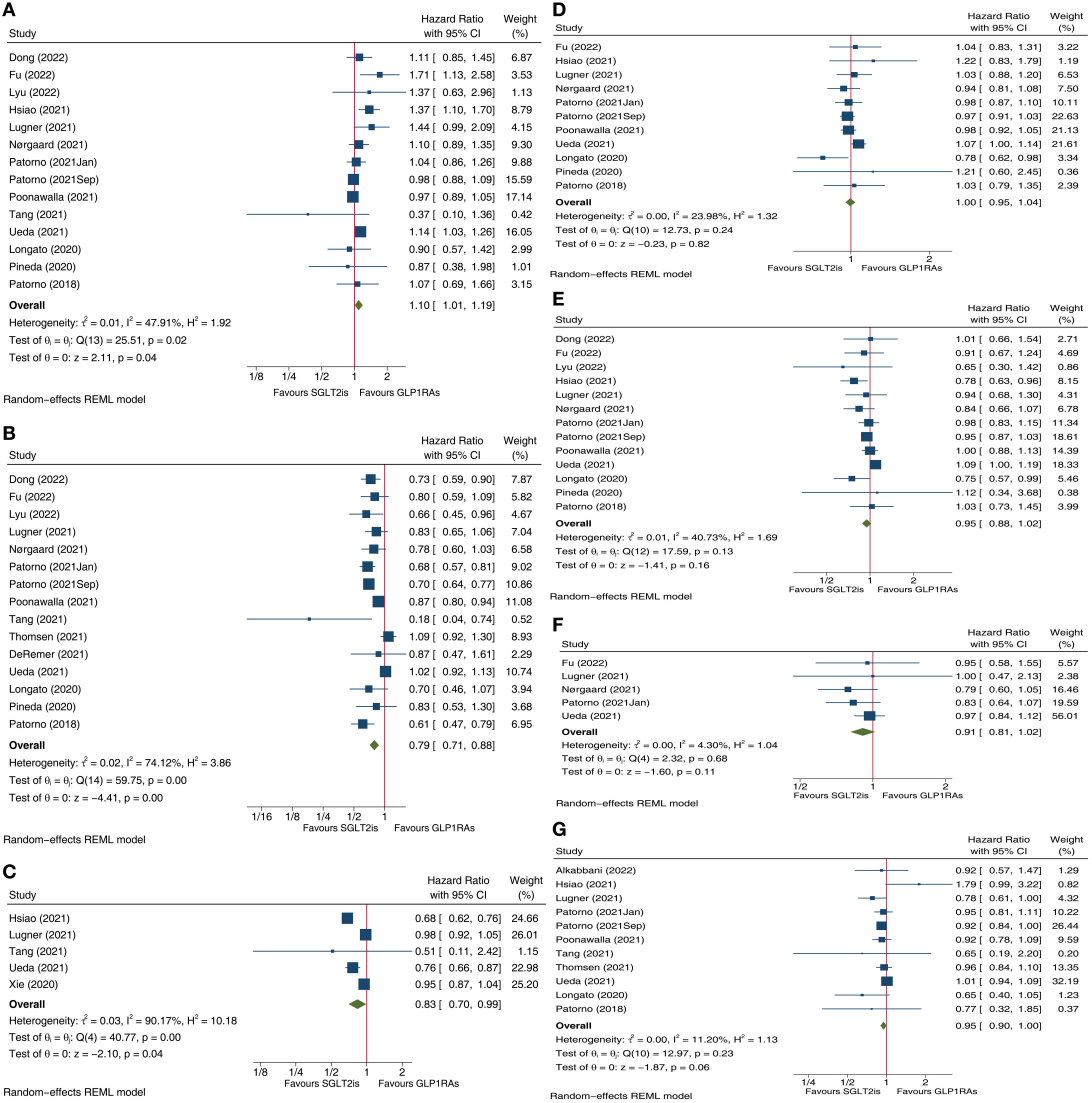
Forest plots of meta-analysis on stroke **(A)**, hospitalization for heart failure **(B)**, composite kidney outcome **(C)**, major adverse cardiovascular events **(D)**, myocardial infarction **(E)**, cardiovascular death **(F)**, and all-cause mortality **(G)**. SGLT2is, sodium-glucose cotransporter 2 inhibitors; GLP1RAs, glucagon-like peptide 1 receptor agonists; CI, confidence interval.

In this updated meta-analysis, we included a total of eighteen large cohort studies (2–19). We identified that compared with GLP1RAs, SGLT2is were associated with a 10% increase in risk of stroke (HR 1.10, 95% CI 1.01–1.19; *P* for effect size = 0.04; Figure 1A), a 21% reduction in risk of HHF (HR 0.79, 95% CI 0.71–0.88; *P* for effect size <0.01; Figure 1B), and a 17% reduction in risk of CKO (HR 0.83, 95% CI 0.70–0.99; *P* for effect size = 0.04; Figure 1C). Moreover, SGLT2is and GLP1RAs had no statistically significant differences in risks of MACE (HR 1.00, 95% CI 0.95–1.04; *P* for effect size = 0.82; Figure 1D), MI (HR 0.95, 95% CI 0.88–1.02; *P* for effect size = 0.16; Figure 1E), CVDH (HR 0.91, 95% CI 0.81–1.02; *P* for effect size = 0.11; Figure 1F), and ACM (HR 0.95, 95% CI 0.90–1.00; *P* for effect size = 0.06; Figure 1G).

This updated meta-analysis revealed that SGLT2is vs. GLP1RAs were associated with significantly decreased risks of HHF and CKO and significantly increased risk of stroke in T2D patients, whereas these two drug classes had similar risks of MACE, MI, CVDH, and ACM. Substantially different with these findings, the main findings in the previous meta-analysis (1) by Qiu et al. are that SGLT2is vs. GLP1RAs were associated with significantly decreased risks of CVDH and ACM whereas they had similar risks of CKO and stroke. Obviously, our meta-analysis included more studies (a total of 18 studies) than Qiu's et al. (1) meta-analysis did (a total of 9 studies), and accordingly substantially updated Qiu's et al. findings.

Although this meta-analysis is an update for Qiu's et al. (1) meta-analysis, this is the first one that identified the significant differences between SGLT2is and GLP1RAs in risks of HHF, CKO, and stroke by incorporating real-world studies. Moreover, several previous network meta-analyses (21–24) based on placebo-controlled, cardiovascular/renal outcome, randomized trials revealed that SGLT2is were superior to GLP1RAs in reducing HHF and composite renal outcome, GLP1RAs but not SGLT2is could reduce stroke, and no significant differences existed in the other cardiovascular and death outcomes between them. Obviously, these findings are almost completely consistent with our findings. Therefore, our findings would further help to select between SGLT2is and GLP1RAs for prevention of HHF, CKO, and stroke in T2D patients. Concretely speaking, SGLT2is should be considered over GLP1RAs as for preventing heart failure and renal failure events, whereas GLP1RAs should be considered over SGLT2is as for preventing stroke. What is worth mentioning, although it is important to select the best option upon different patient characteristics and prevention of different cardio-renal outcomes, both of the two drug classes remain the best therapeutic option for T2D patients due to their long-term cardio-renal benefits (25). Due to the absence of original individual patient data, we failed to explore the impact of relevant patient characteristics (e.g., age, sex, and history of cardio-renal disease) on the relative efficacy of SGLT2is vs. GLP1RAs. These possible modifying factors need to be tested in future research. Another limitation of this meta-analysis is only assessing total stroke but failing to assess ischemic and hemorrhagic strokes, respectively. Further studies filling this knowledge gap are clinically meaningful.

In conclusion, this updated meta-analysis identified that SGLT2is vs. GLP1RAs were associated with significantly decreased risks of HHF and CKO and significantly increased risk of stroke in T2D patients, whereas they had similar risks of the other cardiovascular and death outcomes. These substantially updated the findings of Qiu's et al. (1) meta-analysis, and would further help to select between SGLT2is and GLP1RAs for prevention of specific cardio-renal events in T2D patients.

## Summary

Qiu et al. (1) implemented a meta-analysis based on nine cohort studies directly comparing cardiovascular and renal endpoints between sodium-glucose cotransporter 2 inhibitors (SGLT2is) and glucagon-like peptide 1 receptor agonists (GLP1RAs) in patients with type 2 diabetes (T2D). Nowadays, nine new relevant cohort studies are available and yield some inconsistent findings. Hence, we did an updated meta-analysis by including 18 large cohort studies. We identified that SGLT2is vs. GLP1RAs were associated with significantly decreased risks of hospitalization for heart failure (HHF) and composite kidney outcome (CKO) and significantly increased risk of stroke in T2D patients, whereas they had similar risks of the other cardiovascular and death outcomes. This meta-analysis is the first one that identified the significant differences between SGLT2is and GLP1RAs in risks of HHF, CKO, and stroke by incorporating real-world studies. Our findings substantially updated the findings of Qiu's et al. (1) meta-analysis, and would further help to select between SGLT2is and GLP1RAs for prevention of specific cardio-renal events in T2D patients.

## Author contributions

LD: design. YZ, NX, and CW: conduct/data collection. JQ and DW: analysis. LD and JQ: writing manuscript. All authors approved the manuscript.

## Funding

This work was supported by the Key Laboratory of Neuroimaging, Longhua District, Shenzhen; and Shenzhen Fundamental Research Program (Natural Science Foundations), General Programe for Fundamental Research (Grant No. JCYJ20210324142404012).

## Conflict of interest

The authors declare that the research was conducted in the absence of any commercial or financial relationships that could be construed as a potential conflict of interest.

## Publisher's note

All claims expressed in this article are solely those of the authors and do not necessarily represent those of their affiliated organizations, or those of the publisher, the editors and the reviewers. Any product that may be evaluated in this article, or claim that may be made by its manufacturer, is not guaranteed or endorsed by the publisher.

## Abbreviations

SGLT2is: sodium-glucose cotransporter 2 inhibitors
GLP1RAs: glucagon-like peptide 1 receptor agonists
T2D: type 2 diabetes
HR: hazard ratio
CI: confidence interval
MACE: major adverse cardiovascular events
HHF: hospitalization for heart failure
CVDH: cardiovascular death
MI: myocardial infarction
ACM: all-cause mortality
CKO: composite kidney outcome
PRISMA: Preferred Reporting Items for Systematic Reviews and Meta-Analyses.
